# Hand-held echocardiography during complex electrophysiologic procedures

**DOI:** 10.34172/jcvtr.2023.31621

**Published:** 2023-06-29

**Authors:** Selda Murat, Taner Ulus, Ahmet Serdar Yılmaz, Halit Emre Yalvaç, Ezgi Çamlı, Muhammet Dural

**Affiliations:** ^1^Department of Cardiology, Faculty of Medicine, Eskişehir Osmangazi University, Eskişehir, Turkey; ^2^Department of Cardiology, Kahta State Hospital, Kahta, Adıyaman, Turkey

**Keywords:** Hand-Held Echocardiography, Standard Echocardiography, Electrophysiology, Catheter Ablation

## Abstract

**Introduction:**

Complex electrophysiologic (EP) procedures are time consuming and open to complications. Accurate and rapid recognition of cardiac pathologies is essential before, during, and immediately after such procedures. In this study, we aimed to compare hand-held echocardiography (HHE) with standard echocardiography (SE) to determine whether HHE can be used as a practical and reliable diagnostic tool during such procedures.

**Methods:**

One hundred consecutive patients undergoing complex EP procedures and catheter ablation were included in the study. All patients were evaluated with SE or HHE in terms of main cardiac pathologies at the beginning and immediately after the procedure. The diagnostic accuracy and evaluation time of both methods were compared at the beginning and after the procedure. The agreement between both methods was calculated.

**Results:**

At the beginning and after the procedure, opening and evaluation times with HHE were significantly shorter than with SE (*P*<0.001 for all). There was significant agreement between the two methods in the diagnosis of cardiac pathologies (Agreement was 95% for minimal mild aortic regurgitation (AR), 99% for moderate/ severe AR, 93% for minimal/ mild mitral regurgitation (MR), 95% for moderate/ severe MR, 100% for pericardial effusion, and 100% for left ventricular thrombus at the beginning of the procedure).

**Conclusion:**

With the use of HHE during complex EP procedures, cardiac pathologies can be diagnosed with similar accuracy as SE. In addition, HHE has a significant advantage over SE in terms of time to diagnosis.

## Introduction

Today, thanks to a better understanding of the mechanisms and technological developments, many complex arrhythmias can be treated in electrophysiology (EP) laboratories. The methods used in the treatment of complex arrhythmias are time-consuming and open to the development of complications. Echocardiography is the most widely used imaging technique in EP laboratories to determine such complications. Hand-held echocardiography (HHE), which is smaller, mobile, and easy to use compared to standard echocardiography (SE), is increasingly used in routine clinical practice. HHE has been shown to be applicable in different departments such as emergency room, intensive care units and cardiology outpatient clinics.^1-4^ There is also a need to accurately and quickly identify important cardiac pathologies during complex EP procedures. To our knowledge, there is no data on the use of HHE during complex EP procedures. In this study, we aimed to compare the use of HHE with SE in terms of diagnostic accuracy and evaluation time during complex EP procedures.

## Materials and Methods

 In this prospective, observational, single-center study, 106 patients who underwent complex ablation procedures between March 15, 2020, and August 15, 2021, were consecutively included. Six cases were excluded because of a poor acoustic window and/or insufficient color doppler examination. Thus, 100 patients were included in the final evaluation. Clinical and demographic characteristics were recorded. Complex EP and ablation procedures included those accompanied by a three-dimensional mapping system [Radiofrequency (RF) catheter ablation for atrial fibrillation (AF), typical atrial flutter or non-cavotricuspid isthmus atrial tachycardia, ventricular tachycardia (VT)/ premature ventricular contraction (PVC) in normal hearts or structural heart disease)] or performed by cryoballoon (CB) for AF. All patients gave their written informed consent, and the Local Ethics Committee of our center approved the study.

###  Echocardiographic evaluation

**Figure 1:**
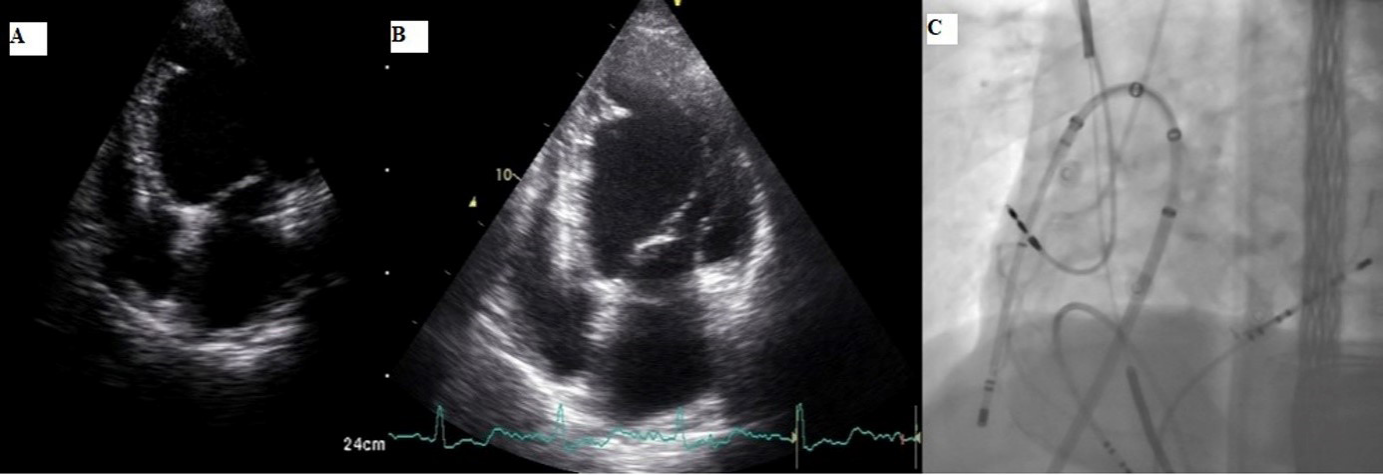
Comparison of post-procedure SE and HHE in a patient who underwent VT ablation with epicardial approach Discriptions: 1A; apical 4 chamber view with HHE, 1B; apical 4 chamber view with SE, 1C; fluoroscopic image of the procedure

All patients were evaluated with echocardiography devices (SE or HHE) in terms of main cardiac pathologies including LV thrombus, mitral regurgitation (MR), aortic regurgitation (AR), and pericardial effusion at the beginning and immediately after the procedure. If there was a hemodynamic disorder, an emergency evaluation was also made during the procedure. HHE (Vscan, GE Vingmed Ultrasound, Horten, Norway) and SE (GE Vingmed Ultrasound with an M3S probe) were performed on all patients by experienced two echocardiographers before and after the procedures. Parasternal long axis and apical four-chamber views were obtained using standard/nonstandard transducer positions. At the same time; nonstandard views were also obtained using apical and low parasternal echocardiographic windows to examine the presence and severity of valvular regurgitation, presence of LV thrombus, or pericardial effusion. The severity of valve regurgitation (0 – none, 1 – minimal, 2– mild, 3 – moderate, 4-severe) was graded according to cardiac morphology and visual interpretation of the color Doppler jet. All measurements were made in accordance with guideline recommendations.^5^ At the same time, the opening and evaluation times of SE and HHE were recorded. In Figure 1, there are echocardiographic images of a patient who underwent VT ablation with the epicardial approach, evaluated with SE and HHE after the procedure.

###  Electrophysiological study, mapping and catheter ablation

Antiarrhythmic drugs were discontinued for at least five half-lives before the procedure. However, if the patient had hemodynamically unstable arrhythmias, the procedure was performed without discontinuing antiarrhythmic therapy. Procedures were performed under conscious sedation unless there was a need for epicardial mapping or hemodynamically unstable arrhythmias that may require electrical cardioversion. Otherwise, general anesthesia was used. A three-dimensional system (EnSite Precision^TM^ Cardiac Mapping System, St. Jude Medical) was used for mapping in procedures other than CB ablation for AF. In cases where the three-dimensional mapping system was used, RF catheter ablation was performed via a contact force sensing catheter (the TactiCath Quartz open-irrigated contact force-sensing catheter). A second-generation 28-mm CB catheter (Arctic Front Advance^TM^, Medtronic) was used for pulmonary vein isolation in cases treated with CB for AF.

###  Statistical analysis

Continuous data were expressed as the mean ± standard deviation for normally distributed variables or as the median [25th, 75th percentiles] for non-normally distributed variables. The Shapiro-Wilk test was used to test whether continuous variables were normally distributed. Paired 2-tailed Student’s t-test and Mann-Whitney U test were used to compare normally distributed and non-normally distributed continuous variables, respectively. Categorical data are expressed as numbers (%) and differences between categorical variables were evaluated using the χ2 test. The agreement between SE and HHE was calculated. Statistical analyses were performed by using IBM SPSS version 24.0 (IBM SPSS Statistics, IBM Corporation, Armonk, New York). A two-tailed *P* < 0.05 was considered statistically significant.

## Results

In 100 patients [age: 56.5 (43.5 – 62.0) years; 63.0% male], 100 consecutive complex ablation procedures were included. The baseline characteristics of the study population are summarized in Table 1. Procedures include CB ablation of persistent/paroxysmal AF (n = 27), RF ablation for atrial tachycardia (n = 11), atrial flutter (n = 11), persistent/paroxysmal AF (n = 11), PVCs (n = 32), VT in structural heart disease (n = 7) or VT in structurally normal hearts (n = 2). Transseptal puncture and subxiphoid pericardial access were performed in 41 and 5 procedures, respectively. In 9 patients, the procedure was performed under general anesthesia. Table 2 lists procedural characteristics.

**Table 1:** Baseline characteristics of study population (n = 100)

Age (years)	56.5 (43.5 – 62.0)
Male sex (n,%)	63 (63.0)
Hypertension (n,%)	38 (38.0)
Diabetes mellitus (n,%)	19 (19.0)
Coronary artery disease (n,%)	17 (17.0)
Heart failure (n,%)	22 (22.0)
LVEF (% )	60.0 (50.5 – 65.0)
Previous ablations (n,%)	4 (4.0)
Presence of ICD/PM (n,%)	13 (13.0)
Haemoglobin, g/dL	14.2 ± 1.7
eGFR, mL/min/1.73m^2^ NP	89.4 (69.7 – 90.0)

LVEF: Left ventricular ejection fraction, ICD: Implantable cardioverter defibrillator, PM: Pacemaker, eGFR: Estimated glomerular filtration rate.

**Table 2:** Procedural data (n = 100)

Ablation type PVCs ablation, n (%) Paroxysmal AF ablation, n (%) Persistent AF ablation, n (%) Atrial flutter ablation, n (%) Atrial tachycardia ablation, n (%) VT ablation in structural heart disease, n (%) VT ablation in normal hearts, n(%)	32 (32.0) 26 (26.0) 11 (11.0) 11 (11.0) 11 (11.0) 7 (7.0) 2 (2.0)
Total procedural time (min)	100.0 (75.0 – 140.0)
Fluoroscpy time (min)	24.5 (15.5 – 35.0)
RF time (min)	13.5 (8.0 – 18.0)
Transseptal puncture requirement, n(%)	41 (41.0)
Subxiphoid pericardial access requirement, n(%)	5 (5.0)
Energy type Radiofrequency ablation, n (%) Cryoballoon ablation, n(%)	73 (73.0) 27 (27.0)

AF: Atrial fibrillation, AV: Atrioventricular, PVC: Premature ventricular contraction, RF: Radiofrequency, VT: Ventricular tachycardia.

###  Echocardiographic results

Details of the echocardiographic examinations are given in Table 3. At the beginning of the procedure, opening and evaluation times with HHE [25.0 (20.0 – 30.0) and 125.0 (120.0 –1 30.0) sec] were significantly shorter than with SE [60.0 (50.0 – 89.7) and 133.5 (130.0 – 143.5) sec] (*P* < 0.001 for both). Again, after the procedure, the opening and evaluation times were significantly shorter with HHE [30.0 (23.5 – 35.0) and 130.0 (122.7 – 135.0) sec] than with SE [57.0 (50.0 – 78.7) and 133.5 (127.2 – 140.0) sec] (*P* < 0.001 and 0.009, respectively) (Table 3).

**Table 3:** Comparison of SE and HHE

	**SE (n=100)**	**HHE (n=100)**	***P*** ** value**
Opening time of device before the procedure (sec)	60.0 (50.0 – 89.7)	25.0 (20.0 – 30.0)	< 0.001
Duration of echocardiographic evaluation before the procedure (sec)	133.5 (130.0 – 143.5)	125.0 (120.0 – 130.0)	< 0.001
Opening time of device after the procedure (sec)	57.0 (50.0 – 78.7)	30.0 (23.5 – 35.0)	< 0.001
Duration of echocardiographic evaluation after the procedure (sec)	133.5 (127.2 – 140.0)	130.0 (122.7 – 135.0)	0.009
**Baseline MR ** Minimal/ mild, n (%) Moderate/ severe, n (%)	63 (63.0) 12 (12.0)	60 (60.0) 13 (13.0)	0.663 0.831
New MR, n (%)	3 (1.0)*	3 (1.0)*	1.000
**Baseline AR** Minimal/ mild, n (%) Moderate/ severe, n(%)	20 (20.0) 4 (4.0)	23 (23.0) 3 (3.0)	0.606 1.000
New AR, n (%)	0 (0)	0 (0)	-
Baseline pericardial effusion	6 (6.0)	6 (6.0%)	1.000
New pericardial effusion, n (%)	5 (5.0)	4 (4.0)	1.000
Baseline LV thrombus, n (%)	3 (3.0%)	3 (3.0%)	1.000

AR: Aortic regurgitation, HHE: hand-held echocardiography, LV: Left ventricle, MR: mitral regurgitation, SE: Standart echocardiography, *: mild degree. *P* < 0.05 statistically significant.

At the beginning of the procedure, MR was detected in 75 patients with SE, and 63 of these (84.0%) were minimal/mild degrees. With HHE, MR was detected in 73 patients at the beginning of the procedure and 60 of them (82.1%) were minimal/mild. Although new MR was observed in 3 patients with both echo devices, it was mild in all of them. There was a significant agreement between SE and HHE results in terms of detecting both minimal/mild and moderate/severe MR (Agreement was 93% and 95%, respectively) (Table 4). At the beginning of the procedure, AR was detected in 24 patients with SE, and 20 of these (83.3%) were minimal/mild degrees. With HHE, AR was detected in 26 patients at the beginning of the procedure and 23 of them (88.4%) were minimal/mild. There was a significant agreement between SE and HHE results in terms of detecting both minimal/mild and moderate/severe AR (Agreement was 95% and 99%, respectively) (Table 5). New AR did not occur in any patient.

**Table 4:** Agreement between SE and HHE for mitral regurgitation

**For minimal/ mild MR**				
		SE		Agreement: 93.0% Sensitivity: 92.0% Specificity: 94.5% PPV: 96.6% NPV: 87.5%
		No	Yes	
HHE	No	35	5	
	Yes	2	58	
**For moderate/ severe MR**				
		SE		Agreement: 95.0% Sensitivity: 83.3% Specificity: 96.5% PPV: 76.9% NPV: 97.7%
		No	Yes	
HHE	No	85	2	
	Yes	3	10	

Abbr: HHE: Hand-held echocardiography, MR: Mitral regurgitation, NPV: Negative predictive value, PPV: Positive predictive value, SE: Standart echocardiography.

**Table 5:** Agreement between SE and HHE for aortic regurgitation

**For minimal/ mild AR**				
		SE		Agreement: 95.0% Sensitivity: 95.0% Specificity: 95.0% PPV: 82.6% NPV: 98.7%
		No	Yes	
HHE	No	76	1	
	Yes	4	19	
**For moderate/ severe AR**				
		SE		Agreement: 99.0% Sensitivity: 75.0% Specificity: 100% PPV: 100% NPV: 98.9%
		No	Yes	
HHE	No	96	1	
	Yes	0	3	

Abbr: AR: Aortic regurgitation, HHE: Hand-held echocardiography, NPV: Negative predictive value, PPV: Positive predictive value, SE: Standart echocardiography

Pericardial effusion was observed in 6 patients with both SE and HHE at the beginning of the procedure. After the procedure, new pericardial effusion was detected in 5 patients with SE and 4 patients with HHE. At the beginning of the procedure, LV thrombus was observed in 3 patients with both SE and HHE. There was a significant agreement between the SE and HHE results in terms of pericardial effusion at the beginning and after the procedure, and LV thrombus (Agreement was 100%, 99% and 100%, respectively) (Table 6).

**Table 6:** Agreement between SE and HHE for pericardial effusion and LV thrombus

**Pericardial fluid at the beginning of the procedure**				
		SE		Agreement: 100% Sensitivity: 100% Specificity: 100% PPV: 100% NPV: 100 %
		No	Yes	
HHE	No	94	0	
	Yes	0	6	
**New pericardial fluid after the procedure**				
		SE		Agreement: 99.0% Sensitivity: 80.0% Specificity: 100% PPV: 100 % NPV: 98.9%
		No	Yes	
HHE	No	95	1	
	Yes	0	4	
**LV thrombus at the beginning of the procedure**				
		SE		Agreement: 100% Sensitivity: 100 % Specificity: 100% PPV: 100% NPV: 100%
		No	Yes	
HHE	No	97	0	
	Yes	0	3	

Abbr: HHE: Hand-held echocardiography, LV: Left ventricle, NPV: Negative predictive value, PPV: Positive predictive value, SE: Standart echocardiography.

###  Complications

 Pericardiocentesis was performed in three patients due to cardiac tamponade developing during or immediately after the procedure. One of these patients was taken to RF ablation because of PVC originating from the right ventricle outflow tract, one was taken to RF ablation for persistent AF, and the other underwent RF ablation for atrial tachycardia originating from the right atrium. Pericardial effusion due to transseptal puncture was observed in first patient, and pericardial effusion developed in the last patient after the third RF ablation procedure. Patients who underwent transseptal puncture were examined with SE, but not transesophageal echocadiography, at 1 month, and residual shunt was not observed. The transient ischemic attack developed in one patient after RF ablation in the aortic cusp region. No phrenic nerve injury or procedure-related death occured. Both the femoral artery and femoral vein accesses were used in 75% of the patients. Access site complications were observed in 4 patients. Among these, 3 were inguinal hematoma, and 1 was a femoral arteriovenous fistula. The patient with femoral arteriovenous fistula underwent surgical repair, and other access site complications were resolved with medical therapy.

## Discussion

 To the best of our knowledge, this is the first study, which evaluates the clinical utility of HHE during complex EP procedures. Our study results demonstrate: 1) There is a strong agreement between the findings of HHE and SE. 2) Both device opening and evaluation times are significantly shorter with HHE than with SE.

In order to minimize the risk during complex EP procedures, it is of great importance to identify findings such as LV thrombus, valve pathologies, or pericardial effusion before the procedure. Again, rapid identification of pathologies such as pericardial tamponade during or immediately after the procedure is very important for early intervention. The use of SE in emergency situations may limited for practical reasons. The use of HHE as a practical tool has been previously reported in a wide range of clinical conditions.^6-13^ A low inter-method variability and interobserver variability were found in previous studies comparing SE and HHE when performed by expert echocardiographers. The sensitivity and specificity of HHE for pericardial effusion were 89-91% and 96%, respectively, and the sensitivity and specificity for valve diseases were reported as 80% and 80%, respectively.^13^

Pericardial effusion is an important complication that can be seen especially after complex RF catheter ablation procedures. Pericardial effusion up to 4.1% and cardiac tamponade up to 1.4% were reported during complex catheter ablation procedures.^14-16^ The number of ablation procedures per patient, additional lesions in addition to pulmonary vein isolation (PVI) during catheter ablation for AF may affect the risk of cardiac tamponade.^15^ The percentage of pericardial tamponade in our study was slightly higher than in previous studies. However, the number of our patients was small. One of the three patients who developed pericardial tamponade were performed additional linear lesions beyond PVI due to persistent AF, and one underwent a third ablation procedure for resistant atrial tachycardia.

Another problem during complex catheter ablation procedures is valve injury. Valve dysfunction may develop for reasons such as the retrograde passage from the aortic valve, antegrade passage from the mitral valve, RF application in the aortic cusp region, RF application in the papillary muscle region.^17^ In our study, the frequency of valve dysfunction related to such procedures was similar to previous studies.^17^ An important condition that should not be missed before complex ablation procedures is the presence of LV thrombus. Subxiphoid epicardial access was performed for VT ablation in 3 patients due to LV thrombus in the study.

While HHE is useful in many clinical areas, it should not be forgotten that it has some limitations and disadvantages. HHE has lower resolution, limited ultrasound frequencies and image optimization in comparison with SE. Also, the screen size is smaller than SE and there is no spectral doppler. Another disadvantage is the lack of hemodynamic measurements.^13^

 In our study, the accuracy of HHE in diagnosing the presence of LV thrombus, presence of pericardial effusion, and valve dysfunction were similar to that of SE, with great consistency between the results of both tools. In addition, both the opening time of the device and the evaluation time with HHE were significantly shorter than with SE.

## Conclusion

 Hand-held echocardiography is a practical tool that can be used in laboratories where complex EP procedures are performed, due to its easy handling, and fast and accurate evaluation. With the availability of HHE in laboratories where such procedures are performed, important cardiovascular pathologies can be diagnosed more rapidly without loss of diagnostic accuracy.

## Competing Interests

 The authors have no relevant financial or non-financial interests to disclose.

## Ethical Approval

 This study was performed in line with the principles of the Declaration of Helsinki. Permission was taken from Faculty of Medicine, Eskisehir Osmangazi University Ethics Committee with dated 30.03.2020, and No: 80558721-050.99-E.39246. Informed consent was obtained from all individual participants included in the study.

## Funding

 This work has been supported by Eskisehir Osmangazi University Scientific Research Projects Coordination Unit under grant number 202011021.
